# Urologic Characteristics and Sexual Behaviors Associated with Prostate Cancer in an African-Caribbean Population in Barbados, West Indies

**DOI:** 10.1155/2013/682750

**Published:** 2013-02-25

**Authors:** Anselm J. M. Hennis, Suh-Yuh Wu, Barbara Nemesure, M. Cristina Leske

**Affiliations:** ^1^Chronic Disease Research Centre, The University of the West Indies, Jemmott's Lane, St. Michael, BB11115, Barbados; ^2^Department of Preventive Medicine, Stony Brook University, Stony Brook, NY 11794-8036, USA; ^3^Ministry of Health, Frank Walcott Building, Culloden Road, St. Michael, BB14001, Barbados; ^4^Faculty of Medical Sciences, Cave Hill Campus, The University of the West Indies, St. Michael, BB11000, Barbados

## Abstract

Prostate cancer (PC) is the principal malignancy affecting African descent men in the Caribbean and the USA. Disparities in incidence, prevalence, and mortality in these populations are poorly understood. We evaluated the urologic characteristics and sexual behaviors of men with histologically confirmed PC (cases) and age-matched controls in the nationwide Prostate Cancer in a Black Population (PCBP) study conducted in Barbados. Cases were around 1.5 to 3 times more likely to report symptoms of prostatic enlargement, hematuria/hematospermia, and previous prostatitis. Sexually transmitted infections (STIs) were similar among cases (24.5%) and controls (26.7%). First sexual intercourse before the age of 16 was associated with an increased likelihood of both low- (Gleason score < 7; OR 1.63; 95% CI: 1.03–1.66) and high-grade PC (Gleason score ≥ 7; OR 1.82; 1.11–2.99). PC risk decreased with later age of sexual debut (P-trend = 0.004). More lifetime sexual partners was associated with increased odds of high grade PC (P-trend = 0.02). The contribution of sexual behaviors to the development and the outcomes of PC is likely due to multiple mechanisms, and further study will be necessary to elucidate the underlying pathophysiologic mechanisms in this and similar populations.

## 1. Introduction

Prostate cancer (PC) rates and mortality are highest among African American men globally [1]. PC is also the most frequent malignancy affecting African-Caribbean men [2–5], a group which shares a common heredity with African Americans as a historical consequence of the West African slave trade. The reasons for the high PC risk and poor outcomes in African-derived populations remain unknown and are likely multifactorial.

Among environmental factors associated with PC, various investigators have described associations between increased PC risk and sexual behaviors [6–8]. In spite of the high burden of PC in the Caribbean, there are few data on disease manifestations or environmental risk factors, particularly sexual behaviors [9, 10]. While some urologic signs and symptoms are commonly observed among persons with PC, this information remains largely unreported in African-Caribbean populations. This paper describes urologic characteristics of men diagnosed with prostate cancer and presents new information about sexual behaviors and PC risk in a national case-control study conducted in Barbados, West Indies.

## 2. Materials and Methods

### 2.1. Study Population

The Prostate Cancer in a Black Population (PCBP) study was a nationwide case-control study of Barbadian men with histologically confirmed newly diagnosed primary PC. Study methods have been described in detail elsewhere [11]. In summary, cases were identified from histological specimens of confirmed prostate cancer evaluated at the country's only Pathology Department, located at the Queen Elizabeth Hospital, Bridgetown. Cases were recruited from men identified with PC between July, 2002 and January, 2011. Age-matched controls (by 5-year age group) were randomly selected from an electronic database of Barbadian citizens and permanent residents and were frequency matched to cases. Of the 1,260 and 1,333 eligible cases and controls, 80% (1,007) and 75% (1,005) participated, respectively. There were no significant age differences between participants and eligible nonparticipants. All study subjects provided informed consent and study protocols conformed to the Declaration of Helsinki. Ethical approval was granted by the Barbados Ministry of Health Medical Ethics Committee and the Stony Brook University's Committee on Research Involving Human Subjects (CORIHS).

### 2.2. Data Collection

Data collection was conducted by trained certified nurse interviewers, masked to the case-control status of participants. They administered a comprehensive standardized study questionnaire, which sought information about participant demographic details, medical and family history, and lifestyle factors prior to the date of PC diagnosis for the cases (and a similarly assigned date for the matched controls). Anthropometric, blood pressure, and pulse rate measurements were also performed by nurse interviewers, and blood samples were collected for assay of HbA1c, PSA, Duffy blood groups, and relevant genetic markers. 

The PCBP study was funded by the National Human Genome Research Institute and the Office of Minority Health, with subsequent funding provided by the National Cancer Institute of the National Institutes of Health. The study's organizational structure comprised a Coordinating Center (Stony Brook Medicine, Stony Brook, NY, USA), a Clinical Center and Local Laboratory Center (Ministry of Health and The University of the West Indies, Bridgetown, Barbados), a Collaborating Center at the National Human Genome Research Institute (Bethesda, MD, USA), and a Gene Discovery Center (Translational Genomics Research Institute, Phoenix, AZ, USA).

### 2.3. Statistical Analyses

Urologic characteristics and sexual risk behaviors investigated in this study were based on self-reported history. Urologic characteristics included previous vasectomy, prostatectomy, and benign prostatic hyperplasia (BPH, and related symptoms of nocturia, urinary retention, dysuria, hesitancy, urgency, frequency, and weak urinary stream), as well as symptoms of painful ejaculation, hematuria, and hematospermia. Sexual risk behaviors were assessed according to the reported history of sexually transmitted infection (STI), age at first sexual intercourse, and lifetime number of sexual partners. Tumors were histologically graded according to Gleason score as low grade disease (score < 7), and high-grade disease (score ≥ 7). Differences between cases and controls were evaluated using *t*-tests (for continuous variables) and chi-square tests (for categorical variables). Odds ratios (ORs) and 95% confidence intervals (CIs) were determined by logistic regression analyses adjusted for demographic and other factors associated with PC in this population (e.g., age, marital status, religion, lifetime occupation, family history of PC, and waist-hip ratio) [11]. The Statistical Analysis System (SAS Institute Inc., Cary, NC, USA) was used to conduct the analyses. 

## 3. Results

Of the total 2,012 PCBP participants, 95% (*n* = 1904) self-reported their race as black or black/white mixed. Due to the small number of white/other participants in the study, this report is based on the 963 incident cases and 941 age-matched controls of African origin. The mean (±SD) ages of the cases and controls were similar: 67.2 (±9.0) years and 67.0 (±9.2) years, respectively [11].

Table 1 presents urologic characteristics of African-Barbadian cases and controls. Vasectomy was rare in both groups and reported by 1.5% and 0.7% of cases and controls, respectively. Multivariate-adjusted odds ratios showed statistically significant differences between cases and controls for symptoms consistent with prostatic enlargement. Cases were between one and a half to three times more likely to report a history of benign prostatic hyperplasia (BPH), nocturia, urinary retention, hesitancy, urgency, frequency of urination, or poor urinary stream. Men with PC were also three times more likely than age-matched controls to report a history of prostatitis (OR; 95% confidence interval: 3.16; 1.92–5.19). Similarly symptoms of dysuria, painful ejaculation, and hematuria/hematospermia were higher in cases than controls, with adjusted odds around 1.7. 

Table 2 presents data about sexual activity reported by cases and controls. Around a quarter of cases (25.4%) and controls (26.7%) reported a previous sexually transmitted infection (STI). While the median age of first sexual intercourse was 17 years in both groups, more men with PC reported their sexual debut at a younger age (*P* = 0.01). The overall number of lifetime sexual partners reported by cases and controls were similar (means of 12 and 10.5, resp.; *P* = 0.28; median of 6). 

Table 3 presents logistic regression results of associations between PC grade and sexual behaviors. Gleason scores were available for 914 cases (data were not located for 49 individuals). A total of 480 men with PC (52.5%) had low-grade disease, and 434 (47.5%) had high-grade disease. For men diagnosed with low-grade PC, first sexual activity before age 16 was associated with a more than 60% increased risk of PC (OR 1.63; 1.03–2.60). Odds ratios for risk of low-grade PC tended to decrease with older age at first sexual intercourse (*P*-trend = 0.06). A stronger relationship was evident for age at first sexual encounter and risk of high-grade PC. First coitus before age 16 was associated with an increased likelihood of high-grade PC (OR 1.82; 1.11–2.99), with a clear trend to a reduced risk with older age at the first intercourse (*P*-trend = 0.02). As expected, the same associations were also evident for analyses based on combined tumor grade (*P*-trend = 0.004). 

With regards to tumor grade and lifetime number of sexual partners, there was no observable trend between number of partners and odds of low-grade PC. There was, however, a statistically significant positive trend between the lifetime number of sexual partners and odds of high-grade PC (*P* = 0.02). The relationship between combined (low and high grade) PC tumors and the number of sexual partners was not found to be statistically significant (*P*-trend = 0.10). Of note, a past history of STI was also not associated with PC tumor grade. 

## 4. Discussion

In this population, cases were between one and a half to three times more likely to report a history of benign prostatic hyperplasia (BPH), nocturia, urinary retention, hesitancy, urgency, frequency of urination, and a poor urinary stream. Men with PC were also three times more likely than age-matched controls to report a history of prostatitis (OR; 95% confidence interval: 3.16; 1.92–5.19). Symptoms of dysuria, painful ejaculation, and hematuria/hematospermia were similarly higher in cases than controls, with the adjusted odds being around 1.7. Approximately 25% of both cases and controls reported a past STI, while more men with PC reported an earlier age at first sexual activity. Men aged less than 16 years at first sexual activity were at an increased risk of both low- and high-grade PC, with a similar relationship evident for high-grade PC in men aged 16–18 years at their sexual debut. There was also a statistically significant trend to a decreased risk of high-grade PC with older age at first coitus. Similarly, higher numbers of lifetime sexual partners was associated with an increased likelihood of high-grade PC.

### 4.1. Urologic Characteristics

Compared to controls, cases reported more symptoms indicative of prostatic enlargement (frequency, urgency, incontinence, poor stream, and urinary retention), as well as features associated with prostate tumors, such as blood in the urine or semen. 

Data from NHANES (2005-2006 and 2007-2008 cycles) indicated that around 1 in 5 American men reported nocturia, a symptom associated with both BPH and PC [12]. The frequency of this symptom was higher in non-Hispanic black men than in other racial/ethnic groups (30.2%, CI 26.7–34.1 versus 20.1%, CI 18.1–22.1, *P* < 0.001). In adjusted models, black race remained an independent predictor of nocturia. An earlier assessment of genitourinary symptoms in the US population carried out in NHANES III assessed lower urinary tract symptoms of nocturia, incomplete emptying, and hesitancy in men aged 30 years and older, as well as symptoms of weak stream and noncancer prostate surgery in men aged 60+. Interestingly, rates of at least 3 symptoms or surgery were similar in black and white men [13]. We report a similar frequency of nocturia (single event nightly) in Barbadian control subjects and the US population (36.1% versus 35.6%), but higher rates in Barbadian men with PC (58.1%). Rates of urinary hesitancy were much higher in Barbadian men with PC compared to control subjects (33.9% versus 21.1%). These frequencies are in contrast with a reported 7.5% prevalence of hesitancy in US men. Barbadian men with PC were approximately 60% more likely to report hematospermia/hematuria compared to controls (*P* < 0.05). While hematospermia is a rare symptom at PC screening in populations with a frequency of 0.5%, around 14% of affected men were later discovered to have PC [14]. This observation highlights the clinical relevance of this symptom.

Few Barbadian men underwent vasectomy, and this procedure was not associated with PC risk. Early studies had suggested possible associations between vasectomy and PC risk [15, 16], but this relationship was likely due to confounding [17].

### 4.2. Sexual Behaviors and PC Risk

Around 30% of Barbadian men with PC reported a first sexual encounter before age 16. This frequency is similar to the experience of cohorts of UK men since 1960, with a “universally reported” median age of first coitus at 16 years, and who also demonstrate a trend to an earlier age of sexual debut over time [18]. The median age of sexual debut in 6 sub-Saharan African countries was somewhat higher and ranged from around 17 to 20 years [19]. The median age of sexual debut among control Barbadian men was 17.8 years, similar to that reported in African populations. Of note, Barbadian men with PC reported their sexual debut at an earlier age (*P* = 0.01). While this finding is suggestive of a possible hormonal etiology [20], there is an evidence that earlier age at first coitus may be associated with more sexual partners, increased risk of STI, and riskier lifestyle behaviors [7, 19, 21, 22], factors all potentially associated with higher risk of PC. 

Our investigation also found statistically significant associations between the lifetime number of sexual partners and high-grade PC among Barbadian men. The relationship between number of sexual partners and PC is not straightforward. Some authors have reported associations between multiple sexual partners and increased risk of PC [7, 8], but this association has not been demonstrated by others [6]. A meta-analysis of case-control studies which investigated PC risk and the number of sexual partners found that PC risk steadily increased from 4% for five sexual partners to 8% for 10 partners and 27% for 30 partners, respectively [10]. PC risk also increased with frequency of sexual activity, such that men who were sexually active once a week had a 6% increased risk, in contrast to a 53% increased risk for men reporting daily activity. In a study of younger men (aged 40–64 years), Rosenblatt et al. [7] also reported increased PC risk associated with multiple female sexual partners. Stratifying by tumor aggressiveness (“aggressive PC” was defined as tumors with a Gleason grade of 8–10 or regional or metastatic disease), this relationship appeared to be stronger for men with nonaggressive tumors. A marginally significant trend of increasing risk was found in men with aggressive PC, which could have been a matter of statistical imprecision due to a smaller sample size than that of the group with nonaggressive tumors.

Hayes et al. [6] examined PC risk factors in a case-control study of black and white Americans. Consistent with national data, they documented higher self-reported rates of syphilis or gonorrhea in African American men with PC compared to white men (18.7% versus 2.5%). The odds of self-reported STI were similar in black (OR = 1.7; 95% CI: 1.2–2.2) and white men (OR = 1.6; 95% CI: 0.8–3.2) with PC, compared to racially matched controls. Encounters with prostitutes and lack of condom use were also associated with increased PC risk. Serological evidence of previous syphilis was also positively associated with increased PC risk.

STIs are postulated to increased PC risk through inflammatory changes, with chronic *in situ *inflammation thought to underpin prostate carcinogenesis [23]. Associations with sexually transmitted diseases and/or specific diseases such as syphilis and gonorrhea have been reported in some studies [6, 7]. We however, did not note any differences in self-reported frequency of STI and PC among cases and controls (24.5% and 26.7%, resp.) in the PCBP study. Under reporting of STIs, however, is a possibility in this population. Participants were asked whether they “have or had a sexually transmitted disease (e.g., gonorrhea, syphilis, chlamydia, etc.)” but the occurrence of specific diseases was not recorded. Therefore, although data were collected to evaluate associations between PC and exposure to infectious agents, it was not possible to evaluate individual exposures in this investigation. It should be noted that while there were no differences between cases and controls in self-reported STIs, we did not measure serological markers of disease. As such, it might be possible that true differences in STI distributions, if present, would not have been detected.

The positive associations between earlier age of sexual debut as well as higher numbers of sexual partners and risk of high-grade prostate cancer might be due to multifactorial exposures such as hormonal factors, chemical and physical injury, inflammatory, and infectious agents leading to cellular injury and later malignant change. However, more research is needed to elucidate these potential risk factors. 

### 4.3. Strengths and Limitations

This large case-control study represents a nationwide investigation that identified all cases of PC occurring in a predominantly African descent Caribbean population, with cases age matched to population-based controls. The study followed standardized protocols and achieved high participation among cases and controls. We described the urologic characteristics reported by cases and matched controls and evaluated the likelihood of cases reporting symptoms compared with controls for the first time in an African-Caribbean population. Men with prostatic disease or symptoms are more likely to seek medical care and to be screened for PC than men who are asymptomatic. In view of this potential for bias, we did not evaluate associations between PC and urologic characteristics. It is also likely that a proportion of men in the control group would have had undiagnosed PC. The net effect of this misclassification would have been to attenuate true associations between risks and observed PC outcomes. While there may be causal relationships, associations between sexual activity and PC risk are invariably subject to recall bias, response bias, and interviewer bias, as well as confounding by factors related to both PC risk and sexual behaviors. For example, persons who practice high-risk sexual activities are less likely to attend routine health exams and are less socially integrated [17]. Questions about a history of prostate symptoms elicited very few “unknown” responses (ranging from 0.1% for urinary retention to 0.5% for weak flow and STI), but a higher percentage of such replies were noted for questions about age at first sexual intercourse and the lifetime number of sexual partners (approximately 15% and 25%, resp.). While recall difficulties and/or cultural concerns might underlie these response limitations, it is difficult to discern whether they would under- or overestimate the associations documented in this study. Nonetheless, we found no differences in the reporting of “unknown” between cases and controls, suggesting that the likelihood of bias would be small, if indeed present. 

## 5. Conclusion

This paper provides the first data about urologic characteristics and sexual behaviors associated with prostate cancer in a Caribbean population. Our findings of increased risk of PC associated with an earlier age at onset of sexual activity and an increased risk of high-grade disease with a larger number of lifetime partners are suggestive of multiple mechanisms contributing to the development of PC in this population. It remains unclear, however, how these underlying processes influence the development of PC and subsequent clinical outcomes. To our best knowledge, this is the first time that such associations have been reported in an African-origin Caribbean population. Further study will be necessary to elucidate the role of potential factors and agents in the pathogenesis of PC in this and similar populations. 

## Figures and Tables

**Table 1:** Urologic characteristics among PCBP African-Barbadian men.

	Cases (*n* = 963)	Controls (*n* = 941)	Multivariate adjusted OR (95% CI)*
Vasectomy, %	1.5	0.7	1.67 (0.65, 4.31)
Prostatitis hx, %	7.8	2.4	3.16 (1.92, 5.19)*
BPH hx, %	44.4	19.8	3.05 (2.46, 3.79)*
Nocturia, %	58.1	36.1	2.41 (1.99, 2.93)*
Urinary retention, %	15.4	6.6	2.60 (1.88, 3.59)*
Dysuria, %	28.0	18.5	1.77 (1.41, 2.22)*
Hesitancy, %	33.9	21.2	1.80 (1.46, 2.23)*
Painful ejaculation,%	4.7	3.1	1.70 (1.03, 2.81)*
Blood in the urine/semen, %	14.9	10.0	1.58 (1.18, 2.11)*
Urgent need to void, %	45.1	33.1	1.64 (1.35, 1.99)*
Frequent urination, %	56.7	41.8	1.72 (1.43, 2.08)*
Weak flow	34.8	21.5	1.85 (1.49, 2.28)*

*OR: odds ratio; CI: confidence interval. *P* < 0.05, based on logistic regression analyses adjusted for age, marital status, religion, lifetime occupation, family history of PC, and waist-hip ratio.

**Table 2:** Sexual behaviors among African-Barbadian men.

	Cases (*n* = 963)	Controls (*n* = 941)	*P* value*
Sexually transmitted infection, %	25.4	26.7	0.54
First sexual intercourse age (years),			
mean ± SD (median)	17.3 ± 3.5 (17.0)	17.8 ± 4.2 (17.0)	0.01
	(*n* = 825)	(*n* = 808)	
<16, %	29.3	24.9	
16–18, %	41.0	41.3	
19–21, %	20.8	21.8	0.01
>21, %	8.8	12.0	
Lifetime no. of sexual partners,			
mean ± SD (median)	12.0 ± 29.0 (6.0)	10.5 ± 19.5 (6.0)	0.28
	(*n* = 707)	(*n* = 708)	
0–3, %	25.0	27.0	
4–9, %	40.7	41.8	
10–20, %	23.5	23.0	0.12
>20, %	10.8	8.2	

*Chi-squaretest for categorical variables; *t*-test for continuous variables.

**Table 3:** Association between prostate cancer grade and sexual behaviors.

	Multivariate-adjusted OR (95% CI)		
	Low-grade PC OR (95% CI)	High-grade PC OR (95% CI)	All PC cases OR (95% CI)
Age at first sexual intercourse (years)^a^			
<16	1.63 (1.03, 2.60)*	1.82 (1.11, 2.99)*	1.74 (1.20, 2.54)*
16–18	1.28 (0.82, 2.00)	1.66 (1.04, 2.66)*	1.43 (1.00, 2.05)*
19–21	1.25 (0.84, 2.18)	1.50 (0.90, 2.49)	1.36 (0.92, 2.00)
>21	1.0 (reference)	1.0 (reference)	1.0 (reference)
	*P*-trend = 0.06	*P*-trend = 0.02	*P*-trend = 0.004

Lifetime no. of sexual partners^b^			
0–3	1.0 (reference)	1.0 (reference)	1.0 (reference)
4–9	1.04 (0.74, 1.46)	1.05 (0.74, 1.48)	1.00 (0.76, 1.31)
10–20	1.21 (0.82, 1.78)	1.16 (0.78, 1.73)	1.14 (0.83, 1.56)
>20	1.09 (0.64, 1.87)	1.74 (1.06, 2.85)*	1.35 (0.89, 2.04)
	*P*-trend = 0.65	*P*-trend = 0.02	*P*-trend = 0.10

Sexually transmitted infection^c^	1.00 (0.76, 1.31)	1.01 (0.77, 1.33)	1.01 (0.82, 1.26)

OR: odds ratio; CI: confidence interval.

*P < 0.05, based on logistic regression analyses adjusted for age, marital status, religion, lifetime occupation, family history of PC, and waist-hip ratio.

^a^Sample size for low-grade PC: 412; high-grade PC: 373; all PC cases: 825; controls: 808.

^b^Sample size for low-grade PC: 349; high-grade PC: 327; all PC cases: 707; controls: 708.

^c^Sample size for low-grade PC: 476; high-grade PC: 426; all PC cases: 948; controls: 930.

## References

[1] Jemal A, Center MM, DeSantis C, Ward EM (2010). Global patterns of cancer incidence and mortality rates and trends. *Cancer Epidemiology Biomarkers and Prevention*.

[2] Hennis AJ, Hambleton IR, Wu SY (2011). Prostate cancer incidence and mortality in Barbados, West Indies. *Prostate Cancer*.

[3] Gibson TN, Hanchard B, Waugh N, McNaughton D (2011). Thirty-year trends in incidence and age-distribution of prostate cancer in Kingston and St Andrew, Jamaica, 1978–2007. *West Indian Medical Journal*.

[4] Bunker CH, Patrick AL, Konety BR (2002). High prevalence of screening-detected prostate cancer among Afro-Caribbeans: the Tobago prostate cancer survey. *Cancer Epidemiology Biomarkers and Prevention*.

[5] Dieye M, Veronique-Baudin J, Draganescu C, Azaloux H (2007). Cancer incidence in Martinique: a model of epidemiological transition. *European Journal of Cancer Prevention*.

[6] Hayes RB, Pottern LM, Strickler H (2000). Sexual behaviour, STDs and risks for prostate cancer. *British Journal of Cancer*.

[7] Rosenblatt KA, Wicklund KG, Stanford JL (2001). Sexual factors and the risk of prostate cancer. *American Journal of Epidemiology*.

[8] Sarma AV, McLaughlin JC, Wallner LP (2006). Sexual behavior, sexually transmitted diseases and prostatitis: the risk of prostate cancer in black men. *Journal of Urology*.

[9] Fernández L, Galán Y, Jiménez R (2005). Sexual behaviour, history of sexually transmitted diseases, and the risk of prostate cancer: a case-control study in Cuba. *International Journal of Epidemiology*.

[10] Dennis LK, Dawson DV (2002). Meta-analysis of measures of sexual activity and prostate cancer. *Epidemiology*.

[11] Nemesure B, Wu SY, Hennis A (2012). Central adiposity and prostate cancer in a black population. *Cancer Epidemiology, Biomarkers & Prevention*.

[12] Markland AD, Vaughan CP, Johnson TM, Goode PS, Redden DT, Burgio KL (2011). Prevalence of nocturia in United States men: results from the national health and nutrition examination survey. *Journal of Urology*.

[13] Platz EA, Smit E, Curhan GC, Nyberg LM, Giovannucci E (2002). Prevalence of and racial/ethnic variation in lower urinary tract symptoms and noncancer prostate surgery in U.S. men. *Urology*.

[14] Han M, Brannigan RE, Antenor JAV, Roehl KA, Catalona WJ (2004). Association of hemospermia with prostate cancer. *Journal of Urology*.

[15] Honda GD, Bernstein L, Ross RK, Greenland S, Gerkins V, Henderson BE (1988). Vasectomy, cigarette smoking, and age at first sexual intercourse as risk factors for prostate cancer in middle-aged men. *British Journal of Cancer*.

[16] Rosenberg L, Palmer JR, Zauber AG (1994). The relation of vasectomy to the risk of cancer. *American Journal of Epidemiology*.

[17] Sutcliffe S, Kawachi I, Alderete JF (2009). Correlates of sexually transmitted infection histories in a cohort of American male health professionals. *Cancer Causes and Control*.

[18] Wellings K, Nanchahal K, Macdowall W (2001). Sexual behaviour in Britain: early heterosexual experience. *The Lancet*.

[19] Marston M, Slaymaker E, Cremin I (2009). Trends in marriage and time spent single in sub-Saharan Africa: a comparative analysis of six population-based cohort studies and nine Demographic and Health Surveys. *Sexually Transmitted Infections*.

[20] Golub MS, Collman GW, Foster PMD (2008). Public health implications of altered puberty timing. *Pediatrics*.

[21] Santelli JS, Brener ND, Lowry R, Bhatt A, Zabin LS (1998). Multiple sexual partners among U.S. Adolescents and young adults. *Family Planning Perspectives*.

[22] Kaestle CE, Halpern CT, Miller WC, Ford CA (2005). Young age at first sexual intercourse and sexually transmitted infections in adolescents and young adults. *American Journal of Epidemiology*.

[23] Wagenlehner FME, Elkahwaji JE, Algaba F (2007). The role of inflammation and infection in the pathogenesis of prostate carcinoma. *BJU International*.

